# 85. Plasma Cell Free Deoxyribonucleic Acid (cfDNA) Fungal Polymerase Chain Reaction (PCR): a Non-Invasive Diagnostic for Invasive Fungal Disease Evaluation in At-Risk Pediatric Oncology and Stem Cell Transplant Patients

**DOI:** 10.1093/ofid/ofac492.010

**Published:** 2022-12-15

**Authors:** Lauren E Kushner, Hayden T Schwenk, Catherine Aftandilian

**Affiliations:** Stanford University, Palo Alto, CA; Stanford University School of Medicine, Stanford, California; Stanford University, Palo Alto, CA

## Abstract

**Background:**

Molecular diagnostics appear promising for early, non-invasive detection of invasive fungal disease (IFD) in immunocompromised patients. Our clinical lab developed and validated cell free DNA (cfDNA) fungal polymerase chain reaction (PCR) assays (Table 1), which have been in clinical use since November 2020 and were recently included in an institutional pediatric clinical care pathway for prolonged febrile neutropenia. We aimed to evaluate the performance of these plasma cfDNA fungal PCR assays in pediatric oncology and hematopoietic stem cell transplant (HSCT) patients with clinical concern for IFD.

**Methods:**

We initiated an observational study of inpatient oncology and HSCT patients who had plasma mold panel (+/- *Candida* panel) cfDNA fungal PCR obtained as part of an IFD evaluation at our freestanding quaternary care children’s hospital. The primary outcome was IFD clinical diagnosis (proven, probable, possible, unlikely) as per EORTC/MSG† definitions within one month of the cfDNA fungal PCR, which was assigned independently by two physicians, with a third physician utilized in cases of discrepancy. Patient demographics, hospital course, imaging results, and clinical laboratory data were abstracted and maintained in an encrypted REDCap© database.

**Results:**

In a preliminary analysis of October 2021-March 2022 data, there were 21 IFD evaluations for 18 patients (Table 2). Most oncology evaluations were for prolonged febrile neutropenia, while many HSCT were non-neutropenic, but on enhanced immunosuppression with new clinical concerns (e.g., respiratory symptoms, persistent fever). Plasma cfDNA detected a mold or yeast consistent with the clinical presentation in 100% of the five proven/probable cases (Figures 1 & 2). All 14 possible IFD cases had a negative cfDNA fungal PCR.

Proven cases are designated with blue icon, while the probable case is orange. All five cases were in oncology patients who did not have history of hematopoietic stem cell transplant. Cell free DNA (cfDNA) fungal polymerase chain reaction (PCR) results include the organism identified, followed by the cycle threshold (Ct) in parenthesis. Maximum Ct for the assay is 45.

Abbreviations: AG: Aspergillus galactomannan index (Ref <0.5); ALL: acute lymphoblastic leukemia.

BAL: bronchoalveolar lavage; 1,3-BDG: 1,3-beta-D-glucan (Ref <60 pg/ml); cfDNA: cell free deoxyribonucleic acid; CNS: central nervous system; EORTC/MSG: European Organization for Research and Treatment of Cancer/Invasive Fungal Infections Cooperative Group & the National Institute of Allergy & Infectious Diseases Mycoses Study Group; L-AmB: liposomal amphotericin B; SARS-CoV-2: severe acute respiratory syndrome associated with coronavirus 2.

**Conclusion:**

Upfront plasma cfDNA fungal PCR successfully detected a relevant mold or yeast with short turnaround time in 100% of cases that were later classified as proven/probable. This appears promising for early, noninvasive diagnosis that enables prompt optimization of antifungal and surgical management, particularly for mucormycosis cases. Additional data may permit consideration of Mucorales agents PCR as a new EORTC/MSG mycologic criteria.

**Disclosures:**

**Lauren E. Kushner, MD**, Roman: Client of my husband's business..

**Table 1:**
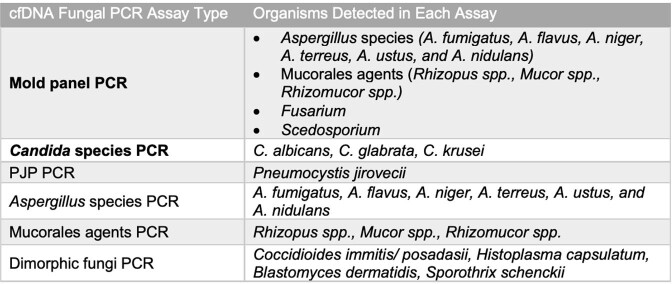
Summary of available cfDNA fungal PCR assays and their targets

Abbreviations: cfDNA: cell free deoxyribonucleic acid; PCR: polymerase chain reaction.

**Table 2:**
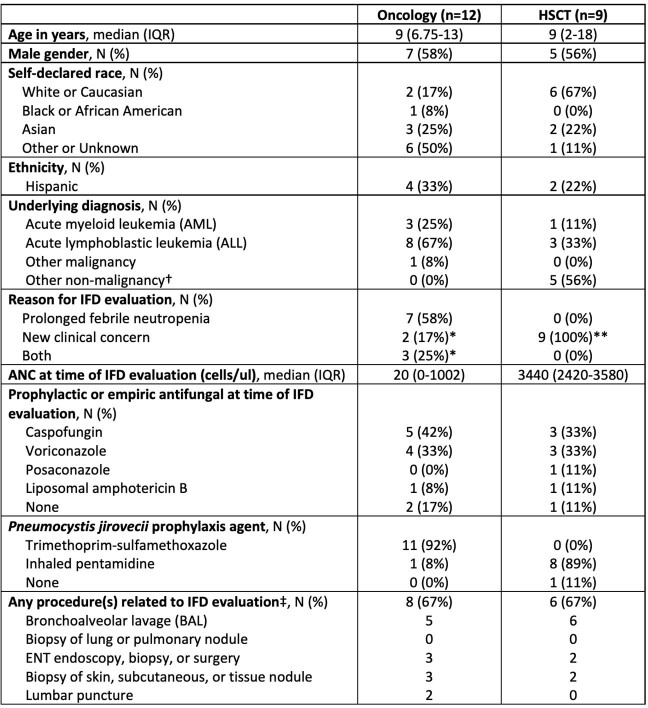
Baseline characteristics for Oncology & Hematopoietic Stem Cell Transplant (HSCT) patient evaluations

†Included hemophagocytic lymphohistiocytosis, hereditary osteopetrosis, STAT1 gain-of-function.

*Oncology clinical concern: 2 (17%) with new oral/sinus concern and 2 (17%) with new respiratory symptoms and new diffuse skin lesions.

**HSCT clinical concern: 5 (56%) evaluations were for new respiratory symptoms and/or oxygen requirement with abnormal imaging. Additional reasons included lab abnormality, new oral/sinus concern, new rash, and persistent fevers in absence of neutropenia.

‡Note: Several patients underwent more than one procedure.

Abbreviations: ANC: absolute neutrophil count (units are cells per microliter, cells/ul); ENT: ear nose throat / otolaryngology; HSCT: hematopoietic stem cell transplant; IFD: invasive fungal disease; IQR: interquartile range.

**Figure 1:**
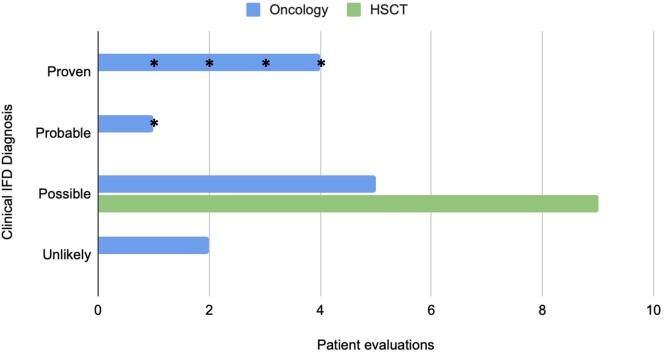
Clinical diagnoses for the 21 patient evaluations for invasive fungal disease by patient group

The cell free DNA (cfDNA) fungal polymerase chain reaction (PCR) was positive in all five proven/probable cases (*), but negative in all remaining cases.

* Designates a patient with positive cfDNA fungal PCR.

Abbreviations: HSCT: Hematopoietic stem cell transplant; IFD: invasive fungal disease.

**Figure 2:**
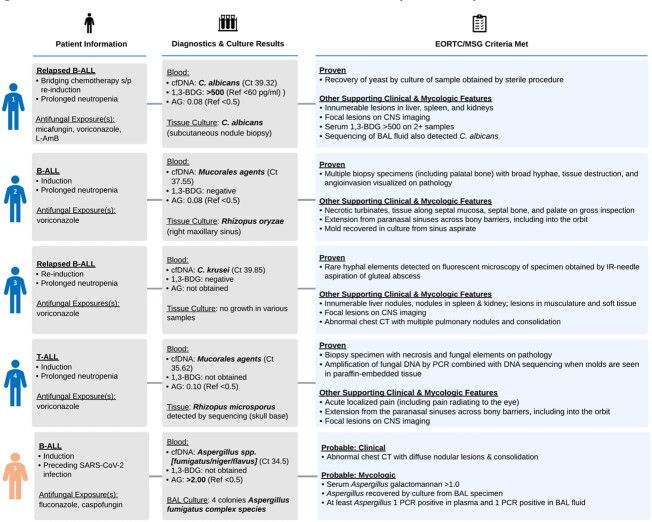
Clinical characteristics of the five proven/probable cases of invasive fungal disease

